# Preclinical immunogenicity assessment of a cell-based inactivated whole-virion H5N1 influenza vaccine

**DOI:** 10.1515/biol-2022-0478

**Published:** 2022-09-26

**Authors:** Zhegang Zhang, Zheng Jiang, Tao Deng, Jiayou Zhang, Bo Liu, Jing Liu, Ran Qiu, Qingmei Zhang, Xuedan Li, Xuanxuan Nian, Yue Hong, Fang Li, Feixia Peng, Wei Zhao, Zhiwu Xia, Shihe Huang, Shuyan Liang, Jinhua Chen, Changgui Li, Xiaoming Yang

**Affiliations:** Viral Vaccines Research and Development Department 2, Wuhan Institute of Biological Products Co., LTD, Wuhan, 430207, China; National Engineering Technology Research Center of Combination Vaccines, China National Biotec Group, Wuhan, 430207, China; National Institute of Food and Drug Control, Beijing, 100050, China; Wuhan Biobank Co., Ltd, Wuhan, 430075, China; China National Biotec Group, Beijing, 100029, China

**Keywords:** MDCK cells, humoral immune response, cellular immune response, H5N1, vaccine

## Abstract

In influenza vaccine development, Madin–Darby canine kidney (MDCK) cells provide multiple advantages, including large-scale production and egg independence. Several cell-based influenza vaccines have been approved worldwide. We cultured H5N1 virus in a serum-free MDCK cell suspension. The harvested virus was manufactured into vaccines after inactivation and purification. The vaccine effectiveness was assessed in the Wuhan Institute of Biological Products BSL2 facility. The pre- and postvaccination mouse serum titers were determined using the microneutralization and hemagglutination inhibition tests. The immunological responses induced by vaccine were investigated using immunological cell classification, cytokine expression quantification, and immunoglobulin G (IgG) subtype classification. The protective effect of the vaccine in mice was evaluated using challenge test. Antibodies against H5N1 in rats lasted up to 8 months after the first dose. Compared with those of the placebo group, the serum titer of vaccinated mice increased significantly, Th1 and Th2 cells were activated, and CD8+ T cells were activated in two dose groups. Furthermore, the challenge test showed that vaccination reduced the clinical symptoms and virus titer in the lungs of mice after challenge, indicating a superior immunological response. Notably, early after vaccination, considerably increased interferon-inducible protein-10 (IP-10) levels were found, indicating improved vaccine-induced innate immunity. However, IP-10 is an adverse event marker, which is a cause for concern. Overall, in the case of an outbreak, the whole-virion H5N1 vaccine should provide protection.

## 1 Introduction

The H5N1 virus is a highly pathogenic avian influenza virus that causes infection and death in poultry and wild birds. It was initially found in Guangdong, China, in 1996 [1]. Humans can be infected by the H5N1 virus in the absence of an intermediate host. In Hong Kong, a total of 18 persons were infected with 6 deaths in 1997 [2]. The clinical signs of the patients were severe pneumonia and infectious multiorgan failure, with fever above 38°C. Upper respiratory symptoms, on the other hand, are not as severe as they are with seasonal influenza [3]. Vaccines are an efficient way to prevent H5N1 outbreaks. In 2020, a phase 2/3 clinical trial verified the safety and efficacy of an H5N1 whole-virion vaccine produced in egg embryos [4]. Although outbreaks of avian influenza might disrupt the availability of egg embryos, cell-based vaccine platforms are able to ensure vaccine production and supply. Easy scale-up, low batch variation, easy control of sterile conditions, and the absence of egg allergens are all advantages of the cell-based platform. Vero cells used to be a common vector for producing the H5N1 vaccine and have been approved by the EU [5,6,7,8]. In recent years, Madin–Darby canine kidney (MDCK) cells have been increasingly used in influenza vaccine manufacturing and are now available in United States, EU, and South Korea. According to reports, India is working on both seasonal influenza and avian influenza vaccines based on MDCK cells [9,10]. In terms of the influenza vaccine market in China, cell-based influenza vaccine has not been licensed in China (including Taiwan) and production capacity of egg-based vaccine cannot meet market demand, so cell-based influenza vaccine could fill a gap in the market. There are two main lines of MDCK cells, suspended and adherent MDCK cell lines (sMDCK and aMDCK). The sMDCK (fetal bovine serum [FBS] is not required) could provide greater yield and lower cost than aMDCK cells. Therefore, we acclimated a serum-free culture of sMDCK from above aMDCK for the H5N1 vaccine manufacturing. According to Kongsomros et al., the H5N1 virus spreads faster and has a greater titer between cells than the H1N1 virus, a feature that has also been found in human lung epithelial cells [11], indicating that MDCK cells might be better suited for H5N1 virus propagation than H1N1 virus propagation. More importantly, eggs might not be suitable for some specific viruses, such as some H3N2 strains, which cannot grow well in eggs, and wild type H5N1, which could kill egg embryos [12]. We cultured H5N1 virus on sMDCK for 72 h in a 40 L bioreactor. The harvested virus was inactivated and purified as a stock solution. Then, we evaluated the immunogenicity of the H5N1 vaccine. We used H5N1 viruses that had been propagated five times in the sMDCK cells for the challenge test because the limited conditions of the laboratory prevented us from using wild strains, and viruses that propagated in sMDCK were pathogenic in mammals. We discovered in earlier experiments that multi-propagation virus could cause significant reduction in body weight and clinical phenomena. Although the vaccine was not supplemented with adjuvants, the viral structural proteins and nucleic acids contained in the whole-virus particles in the vaccine may induce innate and cellular immunity, so the purpose of this study is to comprehensively assess the efficacy of the vaccine.

## 2 Materials and methods

### 2.1 Cells and viruses

sMDCK cells were cultured in VP and DMEM (1:2, with 1% glutamine, bovine serum free). The cells used in the TCID_50_ assay were aMDCK cells from the ATCC, USA. Influenza viruses (A/reassortant/NIBRG-14(Vietnam/1194/2004 × Puerto Rico/8/1934)) were purchased from NIBSC. Working virus seeds were propagated in 9 days specefic pathogen free (SPF) eggs, and the TCID_50_ of the virus was 10^5.67^/0.1 mL. The virus strain used in the challenge test was propagated multiple times in sMDCK cells, and the TCID_50_ was 10^3.5^/0.1 mL.

### 2.2 Vaccines

sMDCK cells were cultured in a 40 L bioreactor (Bio-510, NBS, USA) for 72 h and inoculated with virus prepared in SPF eggs at 33 ± 1°C. To remove host cell protein and DNA, the harvest was clarified by depth filtration (PALL, USA), treated with benzonase, and purified by CaptoTM Core 700 (GE, USA) and CaptoTM Q (GE, USA). β-Propiolactone (Serva, Germany) was added to inactivate the virus. The solution (containing 128 μg/mL hemagglutinin [HA]) was stored at 4°C in phosphate-buffered saline (PBS). The placebo in this study was the PBS that was used to dilute the solution.

### 2.3 Animals

Healthy female BALB/C mice and Wistar rats were supplied by the animal reproduction facilities of Wuhan Institute of Biological Products (WIBP) (experimental animal production license number: 110011216263344054) and kept in the BSL-2 laboratory at the animal facilities of WIBP. The animals were divided into different groups. The grouping details are shown in Tables 1 and 2.

**Table 1:** Animal groups for vaccine injection

Group	Animal species	Purpose	Immunization	Dose group (μg HA/500 µL)	Sampling time (days post first vaccination)	Sampling tissue	Number of animals in each dose group	Total
1	Wistar rat	• Long-term antibody titer	Two doses on days 0 and 28, each delivered IM	0^a^, 1, 2, 3.75, 7.5, 15, 30, 45	28, 42, 56, 84, 112, 140, 168, 196	Blood	5	40
2	BALB/c mice	• Antibody titer		0, 0.5, 1, 2, 3.75, 7.5, 15	28, 42, 56	Blood	10	70
		• IgG subclass^b^						
3	BALB/c mice	• Lymphocyte and cytokine detection		0, 0.5, 1,2, 3.75, 7.5, 15	28, 32, 56	Spleen	5	35
4	BALB/c mice	• Early cytokine production induction	Single dose on day 0, delivered IM	0, 3.75	0^c^, 3, 6, 12, 24, 48 h	Blood	/	/^d^

a: Dose 0 indicates the placebo. Each dose group contained 5 rats; for example, the total number of rats in group no. 1 (rats) was 40.

b: Serum used for IgG detection was obtained from 3.75, 7.5, and 15 μg groups.

c: Samples were collected at hours post-vaccination, and “h” represents hours.

d: The number of animals in this group is detailed in section 2.4.

**Table 2:** Animal groups for the challenge test

Group	Animal species	Treatment before challenge	Sampling time (days post-challenge)	Sampling tissue
5	BALB/c mice	Two doses on days 0 and 28, each delivered IM	6, 8, 10	Lungs


**Ethical approval:** The research related to animal use has been complied with all the relevant national regulations and institutional policies for the care and use of animals, the guidelines of the Laboratory Animal Guidelines for Ethical Review of Animal Welfare (Standardization Administration of China, 2018). Animal studies were approved by the animal ethics committee of WIBP (Ethics No. WIBP-AⅡ332021001).

### 2.4 Challenge test

In the challenge test, the mice were divided into 3 subgroups: a placebo (*n* = 13, 10 for observation and 3 for dissection), a vaccine (same as above), and a blank group (*n* = 2, all for dissection). Mice were injected intramuscularly (IM) with two doses of vaccine (HA 3.75 μg/0.5 mL) or PBS (0.5 mL) on day 0 and day 28. On day 42 (14 days after boost), the mice were challenged with 100 μL of H5N1 virus via the respiratory tract. Body weight and temperature were monitored daily during the challenge phase, day 0–13 post challenge (PC). The mice were checked daily for signs of disease until the test endpoint (day 13 PC). For the dissection mice, the lungs were isolated on day 8 PC and day 11 PC, and the upper lobe of the right lung was sectioned for pathological examination.

### 2.5 Serological tests

The mice and rats were made to bleed via orbital veins. The blood was placed at 37°C for 2 h and centrifuged at 3,000 rpm for 10 min to obtain serum. The sera were stored at −80°C.

#### 2.5.1 Hemagglutination inhibition (HI) assay

The HI assay was conducted based on the China National Influenza Center Standard Practice (Revised Version, 2007). Briefly, the sera were diluted with receptor-destroying enzyme (Sigma, USA), incubated at 37°C for 18 h, and inactivated at 56°C for 1 h. Sera were titrated 2-fold and incubated for 1 h with 4 hemagglutinating units (HAUs) of standard antigen. The initial dilution was 1:10. Then, 1% turkey erythrocytes were combined with the remaining virus. The titers of the HI assay are expressed as the reciprocal of the serum dilution that completely inhibited HA activity at 4 HAUs.

#### 2.5.2 Microneutralization (MN) test

The MN assay was conducted based on the China National Influenza Center Standard Practice (Revised Version, 2007). Briefly, the serum samples were treated as described above. Then, 100 CCID_50_ H5N1 virus was incubated with diluted sera for 1 h at 37°C. Following incubation, the mixture was added to a confluent monolayer of aMDCK cells and incubated for 72 h at 33 ± 1°C. Plates were stained with 1% crystal violet and measured by a spectrophotometer. The titers of the MN assay were considered the reciprocal of the serum dilution that completely inhibited the virus activity.

#### 2.5.3 Subtype classification of IgG

IgG1 and IgG2a subtypes in sera were detected by ELISA kits (Mouse IgG1 ELISA Kit and/Mouse IgG2a ELISA Kit, Bethyl). Dilution and detection procedures were conducted based on the manufacturer’s protocol. Briefly, the plates were precoated with an anti-mouse IgG1 or IgG2a antibody, and diluted samples and standards were added to the plate and incubated for 1 h. After incubation, horseradish peroxidase (HRP)-conjugated antibody, tetramethylbenzidine (TMB) substrate solution, and stop solution were added according to the protocol. The absorbance was read at 450 nm by a microplate reader (Thermo, USA). The concentration of IgG1 or IgG2a in the samples was calculated based on a standard curve.

### 2.6 Intracellular cytokine staining and multiplex cytokine detection assay

Mice were sacrificed and then bathed in 75% alcohol. The spleens were harvested and placed in prechilled medium (RPMI1640, 10% FBS). The spleens were milled individually on a 70 μm cell sieve. The cells were centrifuged (350×*g*, 5 min) and washed with PBS. Lymphocytes were isolated using lymphocyte isolation solution (Dakewe, China), and the lymphocytes were washed with ten volumes of PBS. The lymphocytes were resuspended to 107 cells/mL. H5N1 stock solution was added to 2 mL of lymphocytes. The 2 mL lymphocyte suspension with 5 μg/mL HA was divided equally into 2 parts for 2 tests. The supernatant of 1 mL of lymphocytes was collected 24 h after stimulation for the multiplex cytokine detection assay. The LEGENDplexTM Multi-Analyte Flow Assay Kit was used to detect the concentrations of interleukin-2 (IL)/4/5/6/9/10/13/17A/17F/22, interferon (IFN-γ), and Tumor necrosis factor-α (TNF), following the manufacturer’s instructions. Flow cytometry was performed with a Cytoflex S flow cytometer (Beckman, USA).

Two microliters of Brefeldin A were added to the other 1 mL of lymphocytes after 18 h of stimulation. After incubation for 20 h, the cells were collected for intracellular cytokine staining. In brief, the cells were washed twice with PBS. Then, the cells were stained with 1 μL/test Zombie (Aqua™ Fixable Viability Kit). The cells were sequentially stained with anti-CD3 (APC/Cyanine7 anti-mouse CD3, Biolegend), anti-CD4 (PercpCy5.5 anti-mouse CD4, Biolegend), and anti-CD8 (FITC anti-mouse CD8a, Biolegend) antibodies and incubated for 15 min. Then, 500 μL of fixation solution was added to each sample and thoroughly mixed to fix the cells for 20 min. Then, 1 mL of Perm (1×) was added to wash the cells twice, and then 100 μL of Perm was added to resuspend the cells and permeabilize the cell membrane. Premixed antibodies including anti-TNF-α (APC anti-mouse TNF-α, Biolegend) and anti-IFN-γ antibodies (Brilliant Violet 421™ anti-mouse IFN-γ, Biolegend) were added. The logic order of flow cytometry analysis was Zombie-CD3+ CD4+/CD4+ IFN-γ+ and Zombie-CD3+ CD8+/CD8+ TNF-α+ IFN-γ+. Then, the sample data were obtained from the flow cytometer.

Cytokine changes in the early stage were detected by a LEGENDplexTM Multi-Analyte Flow Assay Kit containing anti-IL-6, anti-IP-10, and anti-monocyte chemotactic protein-1 (MCP) antibodies on flow cytometer.

### 2.7 Clinical observation

Body weight, body temperature, and disease signs, such as back arched, decreased response to external stimuli, and hair erection, were recorded daily from 9:30 to 10:00 a.m. post challenge. All mice were anesthetized with tribromoethanol on day 13 PC.

### 2.8 Virus titer of lung tissue

The amount of lung tissue was weighed, and PBS (1 mg:1 mL) was added. TissueLyser II (Qiagen, Germany) was used to lyse the lung. After lysis, the cells were centrifuged at 800 rpm for 10 min, and the supernatant was collected for titer detection. The supernatant was titrated serially 10-folds until dilution 10^−15^ with VP medium (serum free, 2 μg/mL TPCK-trypsin). Gradient dilutions of homogenized tissue were added to monolayers of MDCK cells in a 96-well plate (initial dilution of 10^−4^) and incubated at 33 ± 1°C for 72 h. Plates were stained with 1% crystal violet and measured by a spectrophotometer. The viral titer in the lungs was estimated by the Reed & Muench Calculator.

### 2.9 Hematoxylin and eosin (H&E) staining and immunohistochemistry (IHC)

Pathology lung sections were prepared as described previously [13]. The steps of H&E staining were as follows. The sections were dipped into a Coplin jar containing hematoxylin and agitated for 30 s. The sections were rinsed in H_2_O for 1 min and then stained with 1% eosin Y solution for 10–30 s, with agitation. The sections were dehydrated with 95% alcohol twice and 100% alcohol twice for 30 s each. Alcohol was extracted with two changes in xylene. One drop of mounting medium was added, and the sections were covered with coverslips.

IHC steps were as follows. The sections were dewaxed, and antigen retrieval was performed using an electric pottery oven. We added 3% hydrogen peroxide to the sections and blocked the sections with mock rabbit serum for 30 min. The primary antibody (influenza anti A/Vietnam/1194/04 (H5N1) HA serum) 1:500, NIBSC, 07/148, UK) was added and incubated overnight at 4°C, and then the secondary antibody (1:500, HRP-rabbit anti-sheep IgG, Biodragon, BF03025, China) was added and incubated for 30 min at 37°C. The sections were rinsed with PBS, and TMB substrate was added. Then, the sections were restained, dehydrated, sealed, and dried. IHC images of sections were collected by a microscope.

### 2.10 Statistical analysis

Prism 9 (GraphPad Software, CA, USA) was used to perform the statistical analysis. *P* values were obtained using unpaired t test or two-way ANOVA nonparametric analysis. *P* values < 0.05 were considered statistically significant. *P* < 0.05 (*), *P* < 0.001 (**).

## 3 Results

### 3.1 The humoral immune response was significantly activated

**Figure 1:**
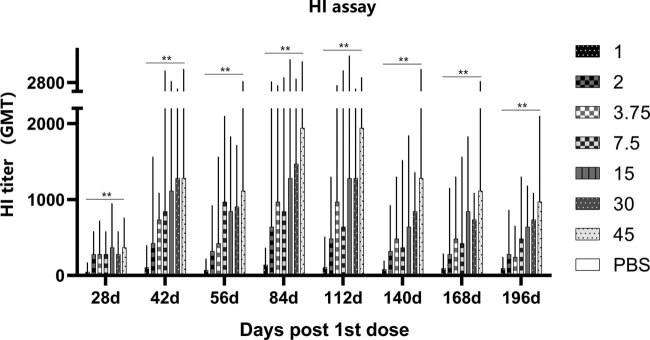
Rats were boosted on day 28. The serum collected after vaccination was used in HI assay. Rats vaccinated with 2–15 μg HA induced strong neutralization antibody responses. Vaccine containing 2 μg HA induced high level antibody in rats for at least 6 months. Statistical analysis was performed using a two-way ANOVA.

The level of antibody secretion is the most direct embodiment of humoral immunity. Specific antibodies in rats can persist for at least 6 months after a boost. The antibody titer was obviously positively correlated with dose (Figure 1). Seroconversion was 100% in all vaccine groups, and antibody titers were far beyond 4-fold change than the placebo group, this scene was not observed for an inactivated quadrivalent split-virus seasonal influenza vaccine based on aMDCK cells (the data are not public). This result suggests that antibody induced by whole-virion particles can provide steady protection to rats for at least half a year.

**Figure 2:**
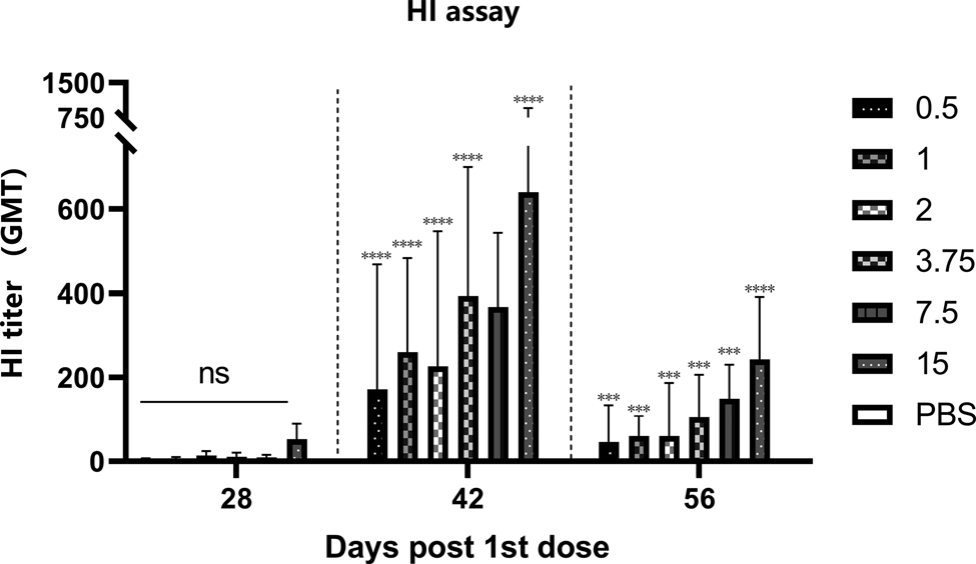
Mouse were boosted on day 28. The serum collected after vaccination was used in HI assay. There is no change in neutralization antibody after first dose. The antibody increased significantly 14 days after boost and decreased significantly 28 days after boost. Vaccine containing 15 μg HA induced highest GMT during the immunization.

**Figure 3:**
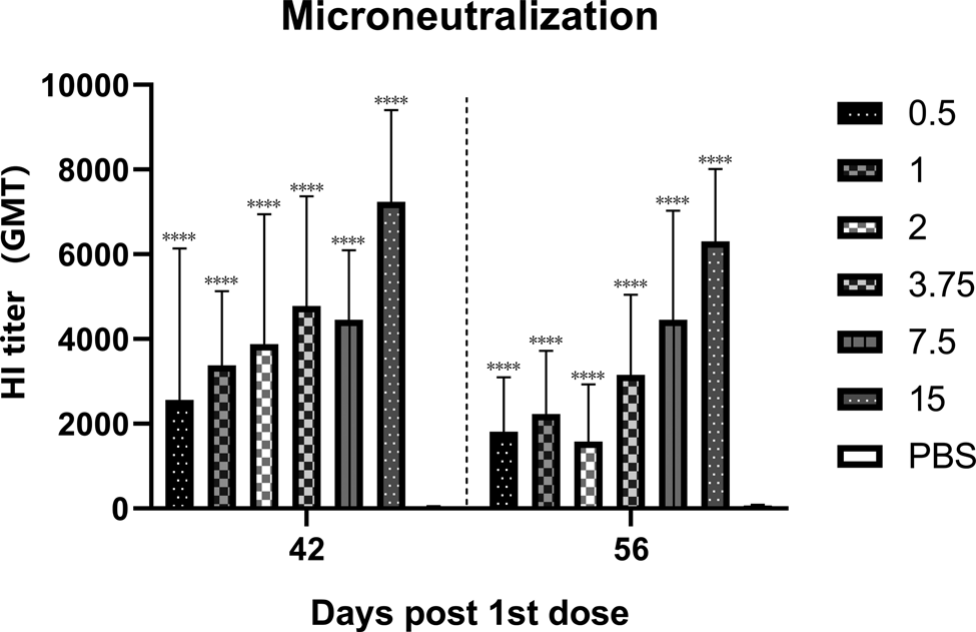
The serum used in microneutralization test were the same as in HI assay. The antibody increased significantly after immunization. There was a decrease in antibody levels, but not as significant as in HI assay.

The antibody titer peaked at day 42 and was at least 4-fold change than the placebo group at day 56 in mice (Figure 2). The MN results in all vaccine groups at 14 and 28 days after boost were also significantly higher than the placebo group (Figure 3). This finding shows that the specific antibodies in serum can effectively neutralize the virus after boost. The HI and MN results showed the same trend, and the geometric mean titer (GMT) increased with increasing vaccine dose. Since the amount of serum at day 28 was insufficient to complete two tests, the MN test for day 28 wase abandoned. We observed significant increases in antibody levels in both rats and mice, suggesting that the vaccine can highly efficiently induce persistent humoral immunity. According to the above results, the MN test showed a higher sensitivity, which may be a better gauge of vaccine effectiveness. Research by Ni et al. on influenza vaccines revealed the same results [14].

### 3.2 The cellular immune response was activated

**Figure 4:**
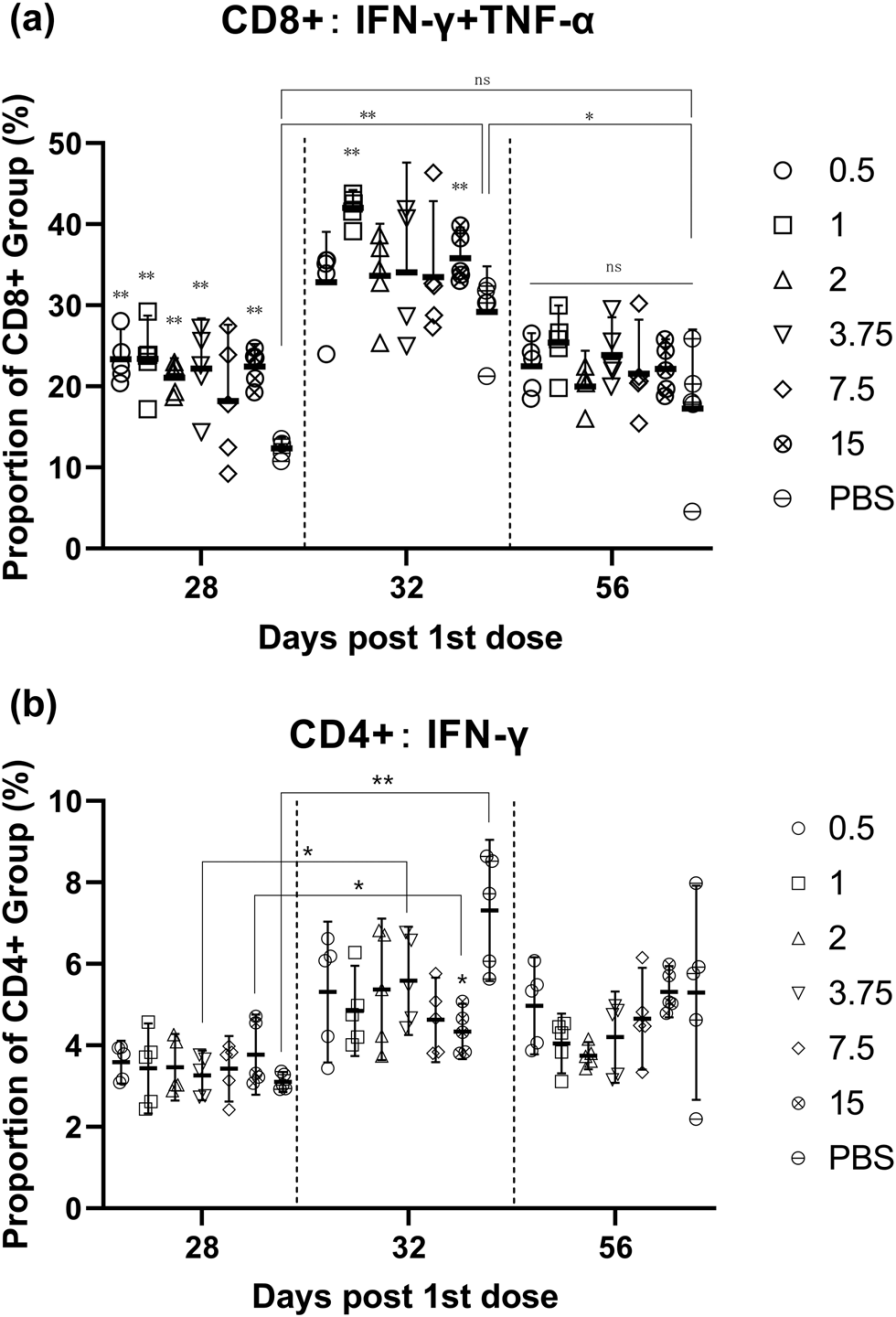
(a) After the first dose, the activated CD8+ T cells were significantly enhanced in the spleen of the mice. The CD8+ T cells were also significantly activated (lower than the 1 ug and 15 ug groups) in the PBS group after boost. All CD8+ T cells decreased to no significant difference in day 56. (b) The differences in CD4+ T cells of splenocytes were not significant at day 28, 32, and 56.

IFN-γ and TNF-α can be secreted by activated CD8+ T cells and therefore used as indirect cytotoxicity markers for activated CD8+ T cells [15]. Both cytokines directly inhibit viral replication and activate immune cells [16]. On day 28, the proportion of CD8+ T cells secreting both TNF-α and IFN-γ in the spleen increased significantly (except for 7.5 μg group). On day 32, the proportion of CD8+ T cells secreting TNF-α and IFN-γ of vaccine groups was still higher than placebo group. On day 56, no difference was observed among the groups (Figure 4a). After the first dose, a large number of CD8+ T cells were activated and specific and nonspecific CD8+ T cells began to participate in the immune response. The changes after boost indicated that virus-specific CD8+ T cells were activated, and specific CD8+ T cells started to proliferate again, forming a population mainly composed of effector CD8+ T cells. After the antigen was removed, the CD8+ T cell population contracted due to apoptosis, leaving only a few memory T cells, and the number of CD8+ T cells returned to normal [17]. The total number of activated CD4+ T cells (IFN-γ+) was unchanged, and the activation was even stronger in the placebo group (Figure 4b).

**Figure 5:**
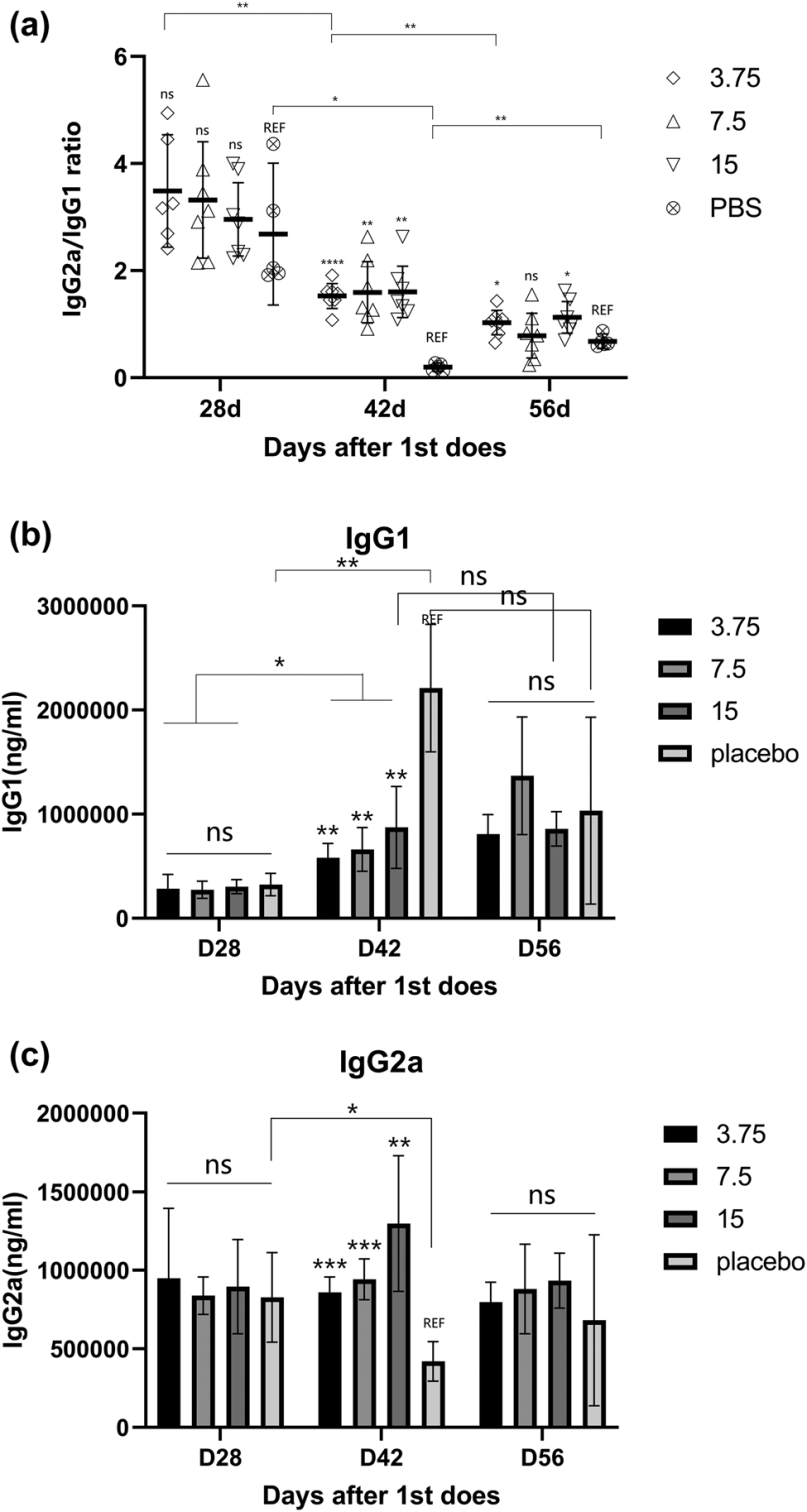
(a) The ratio of IgG2a/IgG1 decreased after boost. The immune response tended to the Th2 direction. This balance remained until day 56. (b and c) There is an antagonism between the Th1 and Th2, both of which showed a significant increase at day 42 after immunization. Both Th1 and Th2 began to exert immune function.

The ratio of IgG2a to IgG1 can be used to describe the direction of the immune response. The subtype classification of IgG indicated the distribution of the Th cell response: IgG2a can represent the Th1 response, and IgG1 is commonly used to represent the humoral immune response. On day 28, there was no difference in the IgG2a/IgG1 ratio between the groups. The IgG2a/IgG1 ratio decreased more significantly in the placebo group than in the vaccine group at day 42, which suggested that boost inhibited part of the decline in IgG2a levels (Figure 5). In addition, there was a significant decrease in IgG2 levels and a significant increase in IgG1 levels in response to placebo stimulation, indicating that Th2 is the dominant immune response. However, the antigens in the vaccine changed this balance, which means that the role of the Th1 response was not diminished or its role was enhanced.

### 3.3 Changes in the levels of cytokines associated with the immune response

**Figure 6:**
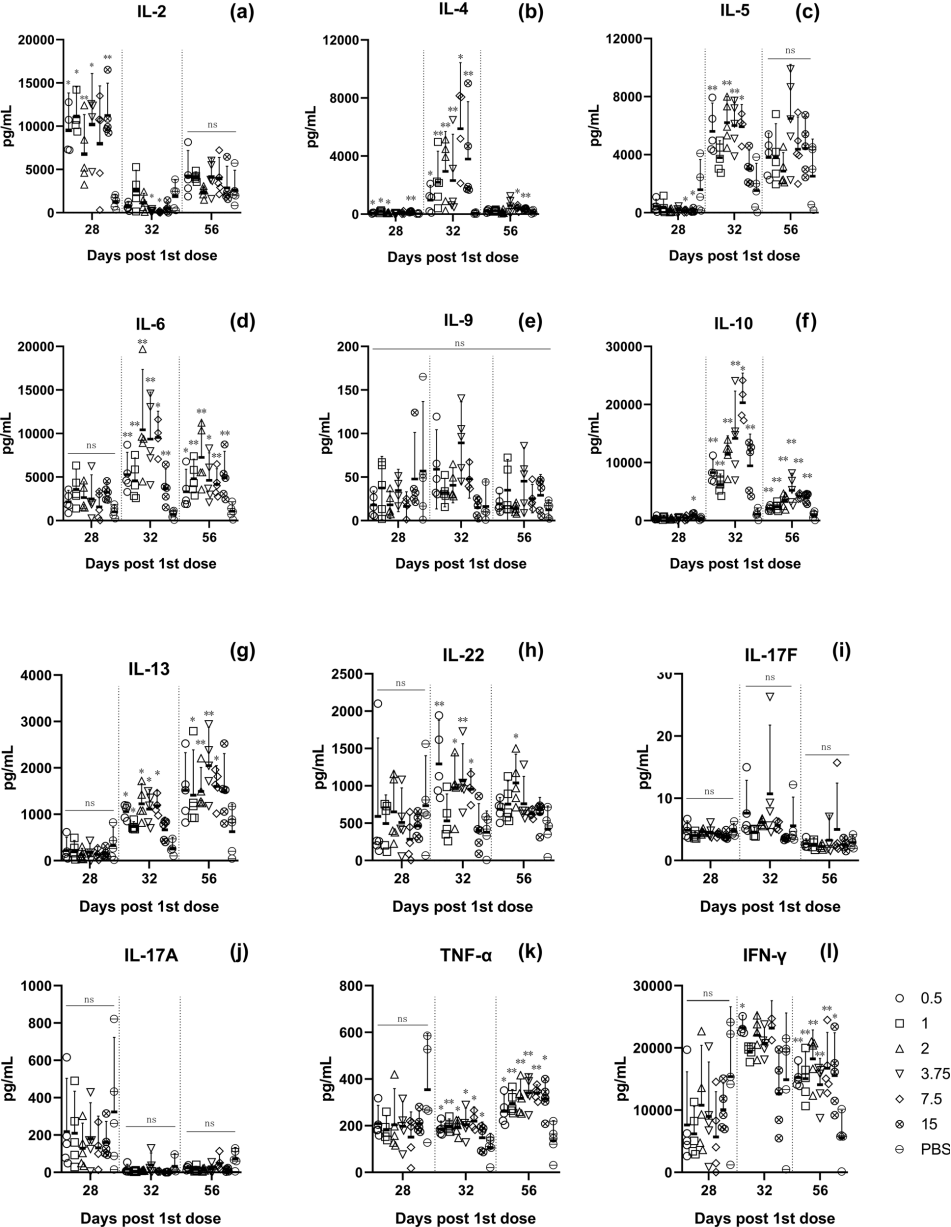
On day 28, IL-2 levels in all vaccine groups (except for 7.5 μg) were significantly higher than those in the placebo group. After booster administration, IL-2 levels in all vaccine groups began to decline on day 32, and there was no difference between the vaccine groups and the placebo group on day 56 (a). IL-4 levels increased significantly on day 32 and declined to the level of the placebo group on day 56, except for those in the 7.5 and 15 μg groups (b). IL-5 levels in the vaccine groups were not different from those in the placebo group on day 28; those in the 7.5 and 15 μg groups were even lower. On day 32, IL-5 levels in the 0.5, 2, 3.75, and 7.5 μg groups were significantly increased compared with those in the placebo group, and on day 56, IL-5 levels in all vaccine groups declined to the level of the placebo group (c). There was no difference in IL-6 levels between the vaccine groups and the placebo group on day 28, but there was a significant increase in IL-6 levels in all vaccine groups on day 32, except for the placebo group. IL-6 levels decreased in all groups on day 56 and were still higher than those in the placebo group (d). IL-9 levels did not change significantly during the immunization period except at a dose of 3.75 μg (e). On day 32, IL-10 levels were significantly increased in all vaccine groups. IL-10 levels decreased in all vaccine groups on day 56 and were still higher than those in the placebo group (f). IL-13 levels increased significantly on day 32 and continuously increased until day 56, but there was no difference between the 15 μg and placebo groups (g). Significant changes in IL-22 were observed at day 28, but gradually decreased with time delay to no significant changes in the placebo group (h). At day 28, there was no significant difference in each group, but IL-17A levels increased after booster administration (j). IL-17F levels did not change significantly during the entire immunization process (i). There was no significant difference in IFN-γ levels between the vaccine groups and the placebo group on day 32 (except for the 0.5 dose group). IFN-γ levels in each dose group were significantly higher than those in the placebo group on days 28 and 56 (l). TNF-α levels in the placebo group were significantly decreased on day 28 and remained stable until day 56, while TNF-α levels in all dose groups were significantly increased at day 56 (k).

To further characterize the immune response, we detected the cytokine content in serum during immunization (Figure 6). These cytokines can be roughly divided into Th1-related cytokines, such as IFN-γ, TNF-α, and IL-2; Th2-related cytokines, such as IL-4/5/10/13; and Th17-related cytokines, such as IL-17A and IL-17F. The secretion level of these cytokines can indirectly explain the activation of relevant cells. IL-2 has an enhanced induction effect on virus-specific cellular immunity [18]. The expression of IL-2 was high before boost, indicating that Th1 cells were going to be activated, while IFN-γ and TNF-α levels did not change. However, after IL-2 levels returned to baseline, the increase in IFN-γ and TNF-α levels became significant, and Th1 cells were activated, which also explained why the decline in Th1 cells was inhibited after boost. The high level of Th2-related cytokines indicated that humoral immunity activation was significant after boost. IL-17A is a hallmark cytokine of Th17 cells [19]. IL-17F and IL-17A are located on the same chromosome, and IL-17F binds to IL-17A to form a dimer [20,21]. Since there were no significant changes in Th17 cell-related cytokine levels, we considered that Th17 cells do not play a critical role in this immune response.

### 3.4 The vaccine provided protection for mice against challenge

**Figure 7:**
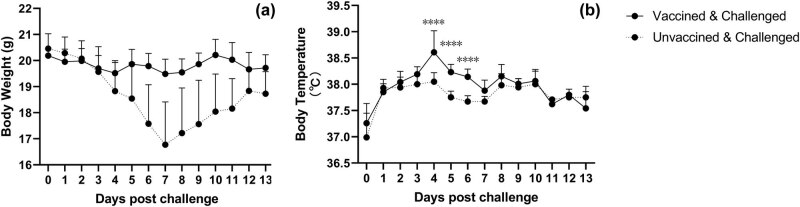
The placebo group began to lose weight at day 4 PC, with the most significant weight loss at day 7 PC (a). Body temperature of the vaccinated group began to rise up to 38.6°C on day 4 PC (b).

In the challenge test, significant weight loss was observed in the placebo group, while in contrast, the vaccine group maintained a stable weight (Figure 7). The change in body weight of the mice in the placebo group occurred on day 4 PC. The highest weight loss on day 7 PC was 30.15%. In the placebo group, eight mice were observed to have significant hair erection and arched back signs, in contrast with 0 mice in the vaccine groups. Significant increase in body temperature was observed in the vaccine group on day 4 PC.

**Figure 8:**
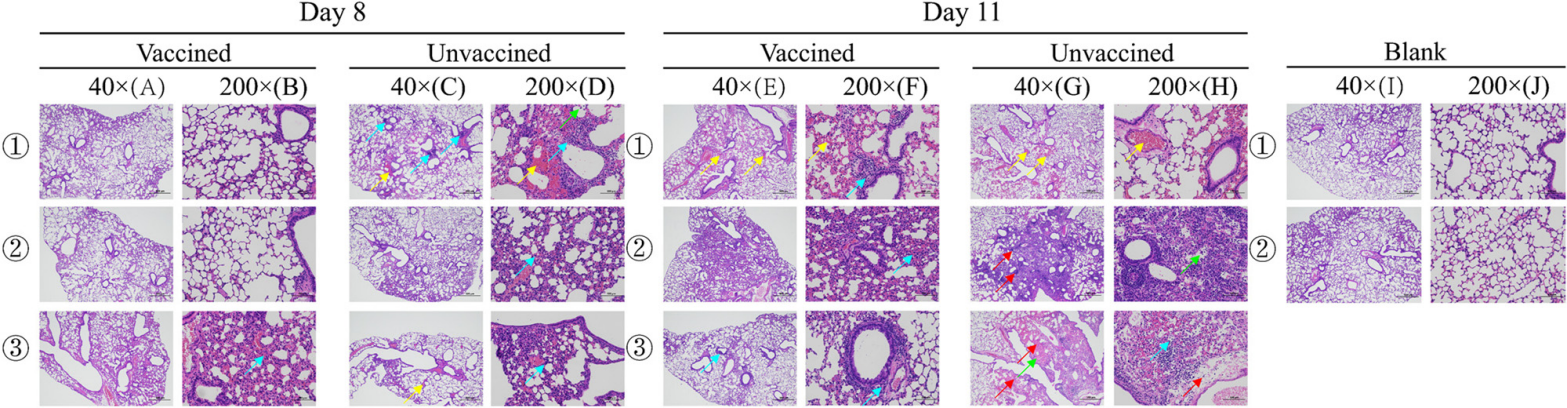
On day 8 PC, few lung lesions in the mice were discovered in the vaccine group, and there were no significant abnormalities in tissues (1A/1B/2A/2B). The alveolar wall was slightly thickened, along with a small amount of granulocyte infiltration (blue arrow) (3A/3B). Significant lesions appeared in the lungs of mice in the placebo group, and it was more common for lymphocytes to show focal infiltration around the blood vessels (blue arrow). Inflammatory cell infiltration was noted in a small number of alveolar cavities (green arrow). Due to local bleeding, red blood cells are visible in the alveolar cavity (yellow arrow) (1C/1D). The alveolar wall was slightly thickened, along with a small amount of granulocyte infiltration (blue arrow) (2C/2D). It was more common that congestion was seen in the blood vessels (yellow arrow). The alveolar wall was slightly thickened, along with a small amount of granulocyte infiltration (blue arrow) (3C/3D). On day 11 PC, lesions also developed in the lungs of mice in the vaccine group. There were multiple congestion sites in blood vessels and alveolar wall capillaries (yellow arrow). Few perivascular lymphocytes showed focal infiltration (blue arrow) (1E/1F). A large area of the alveolar wall was moderately thickened with a small amount of granulocyte infiltration (blue arrow) (2E/2F). Few perivascular lymphocytes showed small focal infiltrates (blue arrow) (3E/3F). However, more severe lesions were observed on day 11 PC in the placebo group, with multiple congested sites in the blood vessels and alveolar wall capillaries (yellow arrow). There were perivascular edema and loose connective tissue, accompanied by a small amount of lymphocyte infiltration (purple arrow) (1G/1H). A large area of alveolar wall was severely thickened, with a small amount of granulocyte infiltration (blue arrow). Inflammatory cells showed diffuse infiltration in multiple alveolar cavities (green arrow). It was more common that perivascular lymphocytes showed focal infiltration (purple arrow) (2G/2H). There was a large area of tissue necrosis (red arrow), with structural disorder. It was more common that eosinophilic homogenates appeared in the alveolar cavities (green arrow), surrounded and accompanied by a small amount of inflammatory cell infiltration. Inflammatory cell infiltration was seen in multiple alveolar cavities (blue arrow). There were perivascular edema and loose connective tissue, with a small amount of lymphocyte infiltration (purple arrow) (3G/3H). No significant abnormalities were found in the lung tissue of mice in the blank group (1I/1J/2I/2J).

**Figure 9:**
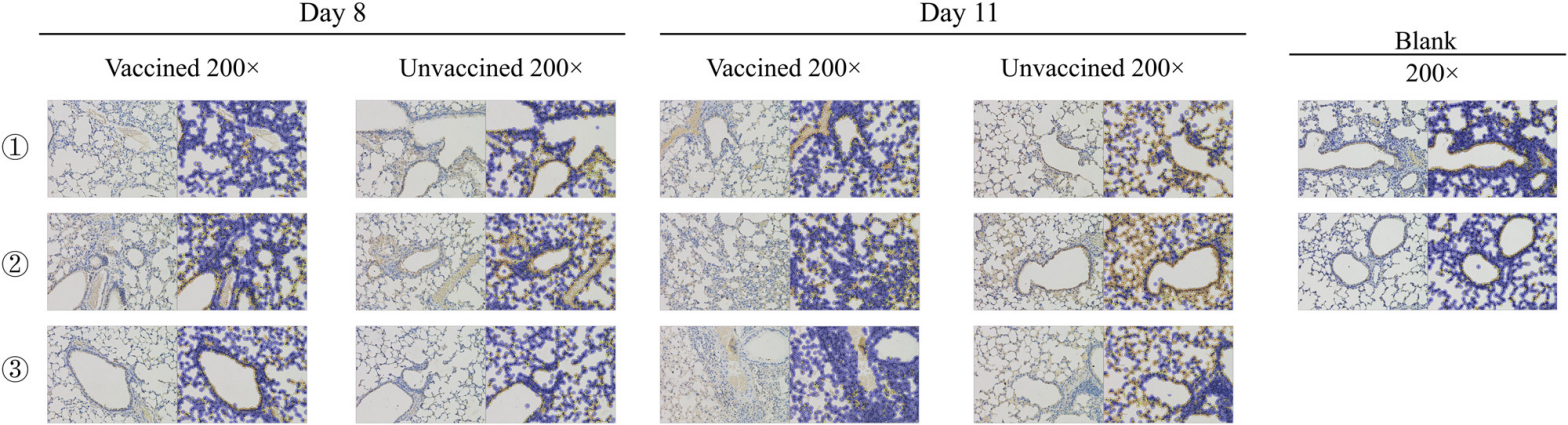
Each image is divided into two parts (left and right). The left part is an image selected from the immunohistochemical slices using a histologic section digital scanner, while the right part is an automatically analyzed left image by Image Analysis System (Servicebio, China). The nuclei are blue and the proteins of H5N1 virus are brownish yellow or dark brown. The positive cell ratio, mean density, histochemistry score, and positive score were calculated.

**Figure 10:**
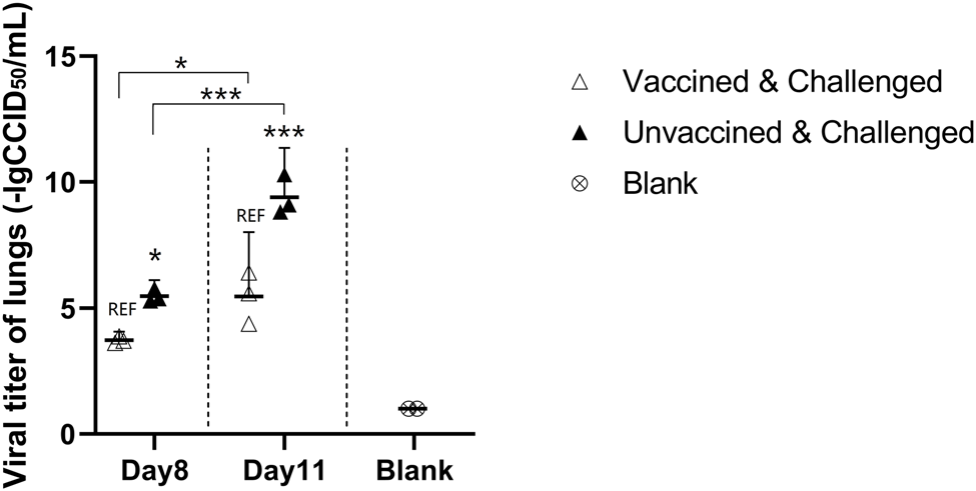
PBS (1 mg:1 mL) was added according to the mass of the lungs. The milled tissue fluid was stored at −80°C after centrifugation. Viral titer testing was performed concurrently after the last sampling. The CCID_50_ results showed that the viral load in the lung was significantly higher in the placebo group than vaccine group.

Significant histopathology in pathology sections of the placebo group was observed (Figure 8). Relatively, histopathology was mild in the vaccine group at the beginning of infection. On day 11 PC, significant histopathology was observed in both the vaccine and placebo groups; however, the extent of lesions in the vaccine group was not as severe as the placebo group. Additionally, the positive cell, mean density, histochemistry score, and positive score results (Table 3) showed that fewer lung cells were infected in the vaccine group than in the placebo group and that smaller areas of lung tissue were infected (Figure 9). The viral titers in the lungs were consistent with these results (Figure 10). Viral titers in the vaccine group were all significantly lower than those in the placebo group during the infection period. Although the above results suggested that the vaccine cannot prevent virus infection in the lungs, it reduces the tissue damage caused by the virus by reducing the virus titer and number of infected areas and cells, thereby reducing clinical symptoms.

**Table 3:** IHC assay

Sample		Positive cell ratio^a^ (%)	Mean density^b^	H score^c^	Positive score^d^	Mean positive score
**Day 8**	Vaccinated	13.74	0.68	19.40	1	2.3
		33.84	0.65	46.17	2	
		30.29	0.68	49.56	4	
	Unvaccinated	39.96	0.67	62.85	4	3.3
		45.21	0.63	74.87	4	
		35.31	0.70	51.24	2	
**Day 11**	Vaccinated	29.60	0.59	43.25	2	1.7
		33.29	0.63	45.64	2	
		10.16	0.66	12.79	1	
	Unvaccinated	76.82	0.72	129.81	8	6.7
		80.76	0.69	144.06	8	
		42.68	0.59	71.61	4	
**Blank**		24.68	0.72	36.77	1	1
		24.06	0.71	35.85	1	

a: Positive cell ratio: (number of positive cells/number of total cells) × 100%.

b: Mean density: number of positive cells/area of tissue.

c: H score: histochemistry score, semiquantitative value of the number of positive cells and staining intensity.

d: Positive score: staining intensity score multiplied by the positive cell ratio score. Score of staining intensity: unstaining presented negative (0 points), pale yellow presented weakly positive (1 point), yellow presented moderately positive (2 points), and brown-yellow presented strongly positive (3 points). Score of the positive cell ratio: 0–5% (0 points), 6–25% (1 point), 26–50% (2 points), 51–75% (3 points), and > 75% (4 points). The larger the positive score is, the stronger the comprehensive positive intensity.

### 3.5 IP-10 levels increased in the early stage

**Figure 11:**
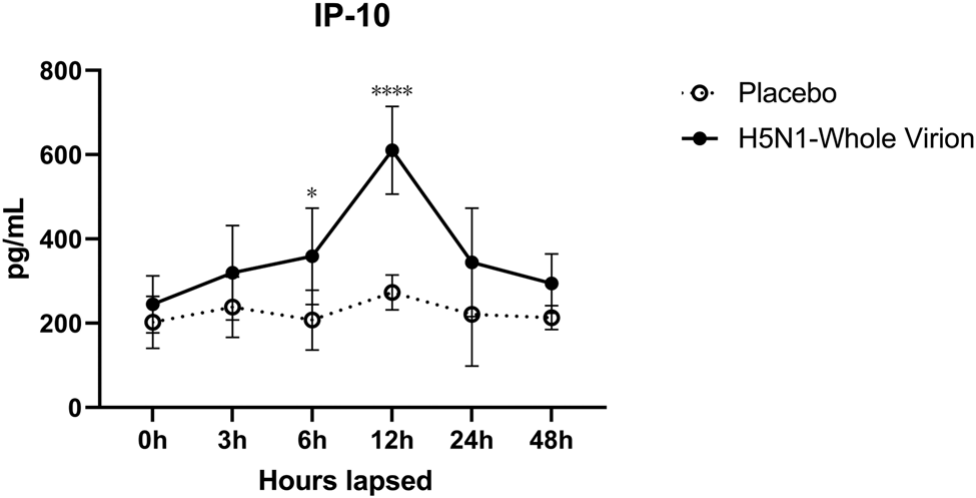
The time for blood sampling was at 0, 3, 6, 12, 24, and 48 h after injection (*n* ≥ 5 per time point). IP-10 appeared to have significant changes at 6 h after injection and reached the peak at 12 h. Then, it dropped to a normal level in 12 h.

More than half of the IL-6 and MCP-1 data were below the limit of detection; therefore, the data were not further analyzed. No significant changes in IP-10 levels were observed between 0 and 3 h after vaccination. The vaccinated group showed a significant change in IP-10 level compared to the placebo group at 6 and 12 h. This difference disappeared in the following 24 and 48 h. IP-10 secretion in the placebo group was consistently stable (approximately 200 pg/mL); on the other hand, the concentration in the vaccine group reached 610.3 pg/mL at 12 h (Figure 11).

## 4 Discussion

Approximately 50% of patients with symptoms after H5N1 infection have died [22]. Although there have not been large outbreaks of H5N1 in the population in recent years, a stockpile of the H5N1 vaccine is essential. Currently, the effectiveness of influenza vaccines of clinical trials are mainly assessed by serological tests, which represent the level of vaccine-induced neutralizing antibodies [23,24]. However, HI assay results mainly showed the binding of specific antibody to antigen. The results of MN experiment revealed the neutralization ability of antibody. Despite the difference in GMT, the results of both tests showed that the vaccine was effective in mice. The two tests conducted at the same time helped us to comprehensively assess the level of humoral immunity in animals. On the other hand, the significant increase in IgG1 content and proportion indicated that Th2 cells were significantly activated after antigen stimulation. Not surprisingly, the levels of Th2-related cytokines such as IL-4, IL-5, IL-10, and IL-13 increased. IL-4, IL-5, and IL-13 are the hallmark cytokines produced in the Th2 immune response and play a key role in immunity against pathogenic microorganisms [25]. Th2 cells also activate macrophages alternatively by expressing IL-4 and IL-13. IL-10 is secreted by Th2 cells to inhibit Th1 cell function [26]. In addition, IL-10 also inhibits chemokines and costimulatory molecules, regulates immunoreaction, and prevents immunopathology [27,28,29]. Th2 cells are activated to help B cells perform their functions. These results indicated that the vaccine induced a high level of specific antibody expression, and humoral immunity began to provide protection. Although the threshold of protection provided by the vaccine is currently expressed as GMT ≥40, the high HI titer cannot guarantee the protection against infection. Indeed, according to the test results, the H5N1 virus can still infect mice with antibody titers higher than 40 and cause lesions in the lungs. There is also a controversy about whether a GMT ≥40 is suitable for the elderly population [30,31,32]. In addition, antigen drift and shift of influenza viruses often cause vaccines to be ineffective against epidemic strains. We therefore focused on the effect of this vaccine on cellular immunity. Some structural proteins and genetic material of live viruses can induce cellular immunity, which can effectively prevent attack by heterologous viruses [33]. IgG2a reflected the activated degree of Th1 cells activation, and IgG1 reflected Th2 cells. The activation of Th1 and Th2 cells reflected the response intensity of cellular immunity and humoral immunity, respectively. So the content of IgG2a and IgG1 reflected the strength of cellular immunity and humoral immunity, while the ratio reflected how the balance of humoral immunity and cellular immunity changes in mice after vaccination [34,35,36]. We discovered that the decline in IgG2a levels was inhibited after boost, indicating that Th1 cells were activated. This result was also supported by a large increase in the levels of TNF-α, which is related to Th1 cells. A mouse experiment showed that CD4+ T cells can provide protection against viruses independent of B cells and CD8+ T cells [37]. Th1 cells can also activate CD8+ T cells and natural killer (NK) cells to help clear intracellular infections [38]. Th1 cells modulate lymph node recruitment of CD8+ T cells, bringing the CD8+ T cells into contact with antigens provided by antigen-presenting cells and thereby activating virus-specific CD8+ T cells [39,40]. In addition to the role of CD4+ T cells, specific CD8+ T cells require antigen activation. Whole-virion particles contain whole viral proteins, which include Matrix Protein, NP, PB1, PB2, PA, etc., all of which are important targets for virus-specific CD4+ and CD8+ T cell recognition. For example, CD8+ cytotoxic T lymphocytes (CTLs) among CD8+ T cells are an important component of cellular immunity in the fight against viral infection [41], and the main function of CTLs is to recognize and kill virus-infected cells by releasing perforin and granzyme B to induce apoptosis [42]. We observed activated CD8+ T cells in the 1 and 15 μg groups. The other dose groups did not show significant differences, but their mean values were higher than those of the placebo group, which might be related to specific proteins of the H5N1 virus. A study on avian influenza showed that highly conserved amino acid residues at residues 58–66 on M1 and 383–391 on NP of the 2009 human H1N1 pandemic virus enhanced the specific recognition by CTLs [43]. M and NP in the H5N1 virus also contain this sequence, so the activation of CD8+ T cells in this study might be caused by those residues. On the other hand, unlike humoral immunity, cellular immunity can provide prolonged protection after vaccination [44,45,46,47]. T cell-mediated immune responses provide a primary/extensive level of protection against influenza infection, and the precise molecular mechanisms behind their protective effects are not known [48]. In summary, after vaccination, both Th1 and Th2 cells are activated and participate in the immune response, and some CD8+ T cells are activated, while humoral immunity is activated and begins to induce the secretion of high titers of neutralizing and long-lived antibodies. The challenge test was a comprehensive assessment of the vaccine’s effectiveness, and the results indicate that the vaccine reduces clinical symptoms in mice. The lungs of the mice were infected in both the vaccine and placebo groups, but the degree of infection was different. The vaccine groups showed no obvious clinical symptoms, i.e., precipitous weight loss, vertical hair, or bowed back. Interestingly, the mice in the vaccine groups developed fever, which may be due to the increased inflammatory response caused by the re-exposure to the antigen in the immunized mice. In conclusion, the vaccine provides protection by reducing symptoms after infection.

In addition, some indicators in the early stage of vaccination are also worthy of attention. Respiratory viruses cause significant changes in the levels of cytokines or chemokines such as IL-6, MCP-1, and IP-10 during infection [49]. IP-10 is widely produced by various cell types on stimulation, including monocytes, T lymphocytes, NK cells, endothelial cells, and stromal cells, among others, where monocytes are responsible for the greatest proportion of IP-10 expression. During the viral infection, the activation of NK cells and monocytes, which function as virus-clearing cells in innate immunity, can be represented by IP-10 expression [50,51]. However, IP-10 is also a marker of adverse event, and could compromise the vaccine’s safety, overexpression of IP-10 can cause acute asthma or other adverse reactions [52]. IP-10 induced by IFN-γ in serum is a biomarker of the severity of acute respiratory infections [53]. It has been shown that the cytokine storm caused by H5N1 may be due to mutations in HA and neuraminidase (NA) glycosylation and sialylation that enhance immune-mediated pathology [49]. Of cause, IP-10 decreased to normal levels after 12 h, suggesting that the acute response may be short-lived. Unfortunately, the changes in IL-6 and MCP-1 levels were not captured in this experiment because the levels were too low. Significant alterations in the levels of all three cytokines generated by an inactivated whole-virion vaccine were detected in C57BL/6 mice in a study by Sekiya et al., which was not observed with a split virus vaccine formulation [54]. Further clinical trials should focus on the dual characteristics of IP-10. The efficacy of the H5N1 vaccine can be further evaluated by a NA inhibition assay. In addition to immunogenicity, the stability and safety of cell-based influenza vaccines also need further study. With the receding of the SARS-COV-2 epidemic, social distance, wearing masks, washing hands, and other measures have also been gradually removed, and a new round of influenza virus outbreaks has also emerged in China [55]. Outbreaks of seasonal influenza viruses such as H3N2 have occurred in several parts of China (https://www.chinacdc.cn/). Although avian influenza viruses such as H5N1 and H7N9 have not been widespread for a decade, they are still evolving in wild birds and could cause a huge social and economic burden once spread among population. Currently, H5N1 vaccines under development are mainly formulated with adjuvants such as MF59 and AS03, which are not yet licensed in China [56,57]. H5N1 vaccines that retain intact virus particles provide long-lasting and cellular immune-inducing protection without adjuvants, and might prevent death from the virus. The results of this study will be submitted to the China Food and Drug Administration for clinical application of H5N1 vaccine.
